# Risks deter but pleasures allure: Is pleasure more important?

**Published:** 2015-05

**Authors:** Li-Wei Chao, Helena Szrek, Rui Leite, Karl Peltzer, Shandir Ramlagan

**Affiliations:** * Population Studies Center, University of Pennsylvania, 3718 Locust Walk, Philadelphia, Pennsylvania 19104-6298, U.S.A.; † Leonard Davis Institute of Health Economics, University of Pennsylvania.; ‡ Porto Business School, University of Porto.; § HIV/AIDS, STIs and TB Research Programme, Human Sciences Research Council.; ¶ Centre for Economics and Finance, University of Porto.; ║ Faculty of Economics, University of Porto.; ** Department of Psychology, University of Limpopo.; †† ASEAN Institute for Health Development, Mahidol University.

**Keywords:** health promotion, health behavior, perceived benefit, perceived risk, risky behavior, smoking, drinking, seatbelt use, sexual behavior

## Abstract

The pursuit of unhealthy behaviors, such as smoking or binge drinking, not only carries various downside risks, but also provides pleasure. A parsimonious model, used in the literature to explain the decision to pursue an unhealthy activity, represents that decision as a tradeoff between risks and benefits. We build on this literature by surveying a rural population in South Africa to elicit the perceived riskiness and the perceived pleasure for various risky activities and to examine how these perceptions relate to the pursuit of four specific unhealthy behaviors: frequent smoking, problem drinking, seatbelt nonuse, and risky sex. We show that perceived pleasure is a significant predictor for three of the behaviors and that perceived riskiness is a significant predictor for two of them. We also show that the correlation between the riskiness rating and behavior is significantly different from the correlation between the pleasure rating and behavior for three of the four behaviors. Finally, we show that the effect of pleasure is significantly greater than the effect of riskiness in determining drinking and risky sex, while the effects of pleasure and riskiness are not different from each other in determining smoking and seatbelt nonuse. We discuss how our findings can be used to inform the design of health promotion strategies.

## 1 Introduction

People do not just pursue unhealthy activities for no reason; they do them for pleasure. Although smoking, problem drinking, risky sex, over eating, etc., have downside risks of morbidity and premature mortality, these unhealthy activities also have the upside of giving pleasure to people who pursue them. Psychologists and economists, trying to explain excessive and harmful risk taking, have developed various models to help explain and predict people’s pursuit of unhealthy activities. These models, in one form or another, embed people’s perceptions of the activity’s costs and benefits. For instance, the Decision Balance Model (Janis & Mann, 1977) emphasizes both the gains and the losses as potential consequences of a decision. The Health Belief Model (e.g., Rosenstock et al., 1988) has at its core the perceived benefit and the perceived susceptibility to illness as well as the severity of illness as determinants of behavior. The Theory of Reasoned Action (e.g., Ajzen, 1991) models positive and negative attitudes about the unhealthy activity as antecedents to intentions to pursue the activity. Subjective Expected Utility Theory models unhealthy behavior as an outcome of a cost-benefit calculation, whereby the costs and benefits are weighted by the subjective perception of the likelihood that these costs and benefits will actually occur (e.g., Gruber 2001; Weber et al., 2002; see Edwards 1954 and 1961 for early reviews).

There is a debate in the literature about whether the pursuit of unhealthy activities is more a consequence of risks not deterring or pleasures alluring the individual to pursue that behavior. Do individuals underestimate the negative consequences, or do they overestimate the anticipated positive consequences?

### 1.1 Background literature

Various studies in the literature try to understand whether people’s pursuit of unhealthy behavior is related to their perceptions of its benefits and risks. Although the methods used to measure these perceptions differ across studies, they all try to elicit the perceived positive and negative consequences associated with various risky activities. For example, Seigel et al. (1994), measuring risk and benefit separately, asked respondents how risky (*and later* how beneficial) it is if they engage in a specific behavior. Benthin et al. (1993), measuring benefit (or pleasure) *relative* to risk, asked: “To what extent are the benefits or pleasures provided by this type of activity greater than the risks associated with it?” Velicer et al. (1985), on the other hand, developed separate instruments with ten items each to measure the “pros of smoking” and the “cons of smoking,” based on the Decisional Balance Model. Using a similar approach, Prochaska et al. (1994) studied the stages of change across twelve unhealthy activities. These studies found support for an association of both perceived benefits and perceived risks with the actual participation in risky activities. These studies, and some others based on more qualitative methods (Dhami & Garcia-Rotemero, 2012; Dhami, Mandel & Garcia-Rotemero, 2011), show that respondents, even youth, can identify and are aware of the different possible benefits and costs associated with various unhealthy behaviors.

A few studies also find perceived benefit to have a greater effect on behavior than perceived riskiness. For instance, Siegel et al. (1994) found that the correlations between benefit and involvement were higher than those between riskiness and involvement amongst college students participating in nineteen risky behaviors. Parsons et al. (1997) found that only ratings of the benefits derived from unprotected sex (but not risk ratings) were significant and negative predictors of consistent condom use as well as significant and negative predictors of stages of change for condom use.

Several studies test the Subjective Expected Utility (SEU) Model by considering not only perceived positive and negative consequences of risky behavior, but also the perceived probabilities that these consequences will indeed occur. For example, Bauman and co-authors showed that the SEU model, using a composite SEU score, explains youth marijuana use (1980), alcohol use (1985), smoking (1984), and sexual behavior (1981). Dhami and Mandel (2012), examining separately the perceived risks, the perceived benefits, and their respective probabilities for health-safety risks, recreational risks, and criminal behavior, found that only perceived benefit was significant in explaining students’ forecast of the chances they would actually undertake a risky activity in the next year, while riskiness (or “drawback”) ratings and the subjective probabilities related to benefits and risks were non-significant. Moore and Gullone (1996) showed that perceived benefit, probability of benefits, and perceived risk were significant predictors for most of the risky behaviors, while probability of risks was not significant for any risky behavior.

The above articles show that the pursuit of an unhealthy behavior is determined in part by how the benefit and the riskiness related to that behavior are perceived. Moreover, the effect of perceived benefit and the effect of perceived riskiness on behavior may differ for different behaviors. In this paper, we ask whether smoking, problem drinking, seat-belt nonuse, and risky sexual behavior are driven by differences in perceptions of the riskiness and pleasure associated with each behavior.

Additionally, we ask whether the effect on behavior from perceived pleasure is statistically different from the effect on behavior from perceived riskiness. Researchers generally test separately for the significance of pleasure perceptions and for the significance of riskiness perceptions in predicting behavior. Although one explanatory variable (pleasure) may be significant and another (riskiness) not significant, that does not necessarily imply that the two variables— pleasure and riskiness—have statistically significantly different effects (e.g., Gelman & Stern, 2006). Everything else equal, an intervention would produce more behavior change if it addressed the factor that has the greater effect on behavior; hence, it is relevant to know whether perceived riskiness and perceived pleasure have statistically different effects on behavior before making any changes in public policy.

Finally, we examine the effectiveness of various health interventions to reduce unhealthy behavior. Using some simplifying assumptions, we compare the effectiveness of three programs: (1) a riskiness-only program that, like most status quo interventions, focuses on increasing perceived riskiness (i.e., to scare the public into reducing unhealthy behavior); (2) a naïve program that expends resources equally to reduce perceived pleasure and to increase perceived riskiness; and (3) an informed program that targets either pleasure or riskiness depending on which factor has statistically greater impact on each behavior.

## 2 Method

### 2.1 Respondents

Respondents (N=351) were recruited during their HIV post-test counseling sessions in one of twelve mostly rural health clinics around Witbank, in the province of Mpumalanga, South Africa. Respondents were referred from another study that recruited a systematic sample of consecutive post-HIV testing clients who visited the study clinics. Every HIV positive respondent and every third HIV negative respondent was recruited, resulting in a sample with almost a 1:1 ratio of HIV positive to HIV negative clients. Participation in our study took place two months after the respondent’s HIV tests, and, due to higher attrition of HIV+ clients, 60% of our sample was HIV negative. Respondents were reimbursed travel expenses and were paid the money earned in the various activities of the study (Szrek et al., 2012). Because we expected some respondents to be illiterate or have low literacy levels and other respondents to be very sick, the questionnaire was privately administered by trained interviewers who went through the survey one-on-one with each respondent. All questionnaires were translated into Zulu, the language of our respondents. Data were collected between September 2009 and April 2010. Our final sample for this paper has N=336, which consists of respondents who answered all the questions related to the four unhealthy behaviors in this paper.

### 2.2 Measures^1^

#### Self-reported actual risky behavior

We measured the respondent’s pursuit in real life of each of the following four unhealthy activities: tobacco use, problem drinking, seatbelt nonuse, and risky sex without a condom.

Tobacco use was defined as daily or almost daily use of any tobacco products, such as cigarettes, snuff, chewing tobacco, or cigars.

For problem drinking, we used the AUDIT-C, which is a three-question version of the Alcohol Use Disorder Identification Test questionnaire (Bush et al., 1998). AUDIT-C measures consumption of alcohol (i.e., the frequency of drinking and the quantity consumed at a typical occasion) and the frequency of heavy episodic drinking. Problem drinking was defined by using an AUDIT-C cut-off score of four for men and three for women (Bush et al., 1998; Gual et al., 2002).^2^

Seatbelt use was assessed by asking the respondent if a seatbelt was used the last time the respondent sat in the front seat of a car.

A respondent was classified as someone who had risky sex if: (i) the respondent did not have a regular partner and did not use a condom during the most recent sexual intercourse; or (ii) the respondent was married/cohabiting, but had more than one partner in the last 12 months and did not use a condom during last sex.

#### Perceived riskiness and perceived pleasure

We measured the respondent’s perceptions of riskiness and pleasure in partaking in four different hypothetical risky activities related to health. The questions were fashioned after Siegel et al. (1994) and Weber et al. (2002). The perceived level of riskiness that the respondent felt was associated with each activity was elicited by pointing to a picture of a scale and asking: “With 1 representing not at all risky and 7 being extremely risky, how risky do you think it is to: *Drink heavily on weekends*.” The italics was replaced by the other health risk behaviors (e.g., smoke half a pack of cigarettes a day, sit in the front seat of a car without a seatbelt, and have sex with a new partner without a condom). Perceived pleasure associated with the activity was elicited by asking: “With 1 representing no pleasure at all and 7 representing extreme pleasure, how much pleasure do you derive from: *Drinking heavily on weekends,*” (and similarly for the other activities). These riskiness and pleasure ratings given by the respondent were then used as the perceived riskiness and the perceived pleasure for the respective activity for that respondent.

#### Demographics

We collected information on age, gender, the highest level of education completed, marital status, the average household monthly income, the importance of religion, and numerical reasoning, which was the number of correct answers to a five-question instrument that assesses the ability to do basic numerical calculations (Szrek et al., 2012).^3^

## 3 Results

Table 1a presents the proportion of participants who pursued various unhealthy behaviors, their risk and pleasure perceptions for activities similar to the behaviors, as well as the correlations between these variables. (The type of correlation coefficient presented is described for this and all other tables in the note below each table.) The means of pleasure ratings range from 1.73 (smoking) to 2.32 (seatbelt nonuse) on a scale of 1 to 7, with riskiness ratings ranging from 6.07 (seatbelt nonuse) to 6.60 (risky sex). Almost a quarter of the respondents are classified as problem drinkers and seat-belt nonusers, while 11% are frequent smokers, and 18% are classified as having recently engaged in risky sex. Although pleasure for all four behaviors is positively correlated with each other and also negatively correlated with riskiness ratings, the correlations between the ratings and the behaviors are quite behavior-specific. For instance, only smoking’s riskiness is related to frequent smoking behavior while riskiness perceived for other activities are not; both pleasure and riskiness ratings for drinking are related to drinking but unrelated to any other behavior; and similarly, seatbelt nonuse is correlated only with its own pleasure and riskiness ratings.

Table 1b presents means for demographics, and the correlations between these variables and actual risk taking. The sample has more women than men, a wide age range from 18 to 71 years, with 42% having completed secondary school or above, and with two-thirds living in households with monthly incomes of 4,000 South African Rand a month or less (approximately $540 USD at the time). Men are significantly more likely to be frequent smokers or problem drinkers than women, while those with a higher education are less likely to be frequent smokers; older adults are more likely to wear a seatbelt, and married individuals are less likely to be identified as engaging in risky sex. Among the four unhealthy behaviors, only smoking and drinking are correlated with each other.

Table 1c presents correlations between ratings and demographic variables. Men are more likely to perceive both binge drinking and risky sex to be pleasurable and smoking to be less risky, while education and income are both correlated with perceiving higher riskiness for smoking and risky sex. Education is also related to perceiving lower pleasure of smoking. Also, people who think religion is very important to them have lower perceived pleasure for binge drinking.

Table 1d shows correlations between the demographic variables. Older respondents are more likely to be married and religious; they also have lower numeracy, education, and income—the latter three variables being highly correlated with each other.

### 3.1 Predicting real-life unhealthy behavior

Table 2 presents the mean pleasure and riskiness ratings for each of the four hypothetical activities, separately for respondents who did and who did not pursue similar behavior in real life. Separate means were calculated for respondents who were classified as smokers, problem drinkers, seatbelt nonusers, or those having risky sex. The difference in ratings between real life risk takers and non-takers was tested for statistical significance controlling for all sociodemographic characteristics. For instance, the second row shows that respondents who were classified as problem drinkers by the AUDIT-C instrument gave a mean rating of 2.79 for their pleasure rating for participating in hypothetical weekend binge drinking. This is statistically significantly higher than the mean of 1.90 rated by respondents who were not problem drinkers. The ratings of the riskiness of binge drinking, however, were not statistically different between the two groups.

We next ran logistic regressions to examine the contribution of both perceived pleasure and perceived riskiness in explaining participation in real life unhealthy behaviors, controlling for the respondent’s gender, age, age squared, educational level, marital status, numerical reasoning, importance of religion, and income levels. Table 3 presents the odds ratios of how much the respondent’s ratings of perceived pleasure and perceived riskiness predict the probability that the respondent pursues a similar risky behavior in real life. (The full regressions are shown in Appendix 2, columns 1 to 4.) It is evident from Table 3 that pleasure and riskiness ratings contribute differently, depending on the actual unhealthy behavior. Respondents who gave lower riskiness ratings for smoking were more likely to be real life daily tobacco users, while their pleasure ratings were non-significant. In contrast, pleasure rather than riskiness predicted problem drinking and risky sex. Finally, both riskiness and pleasure ratings were predictive of seatbelt nonuse in real life.

### 3.2 Comparing significance of correlations

We next ask whether the correlations between pleasure and behavior and riskiness and behavior are statistically significantly different from each other. Because we are measuring behavior from the same individuals, we use the Steiger test of dependent correlations (Steiger, 1980).^4^ We find that the correlations are statistically significantly different for smoking, drinking, and risky sex (Table 4): smoking is more strongly predicted by riskiness; drinking and risky sex are more strongly predicted by pleasure.

### 3.3 Effectiveness of changing perceived pleasure versus perceived riskiness in health promotion

The results thus far suggest that pleasure should not be overlooked in health promotion efforts. Nevertheless, a mere difference in significance between pleasure and riskiness does not imply that the difference between the two measures is significant (Gelman & Stern, 2006). We test this formally, first by examining one behavior at a time, and then by examining all four behaviors simultaneously. We first ran logistic regressions as in Table 3, and then did post-estimation tests to see whether the effect on behavior achieved by a one standard deviation reduction in pleasure differs from the effect on behavior achieved by a one standard deviation increase in riskiness. The results, presented as odds ratios in Table 5a, show that the effects of pleasure reduction had a greater impact on behavior than the effects of increasing riskiness on reducing drinking and risky sex, but are not different for smoking or seatbelt nonuse. Although Table 3 shows riskiness but not pleasure was significant in determining smoking and seatbelt nonuse, the result in Table 5a shows that the effect of riskiness did not differ statistically from the effect of pleasure on smoking or seatbelt nonuse behaviors.

Finally, we use a similar setup with logistic regression and post-estimation tests to examine all four behaviors simultaneously. To make this comparison meaningful and without loss of generality, we make two simplifying assumptions: (1) the cost to increase a unit of perceived riskiness is the same as the cost to decrease a unit of perceived pleasure, and this applies across all behaviors, and (2) the value of unhealthy behavior reduction—such as in the quality adjusted life years gained—is the same across behaviors. These simplifications allow us to focus only on effectiveness.

We compare three different possible programs. The first program (called the riskiness-only program) targets riskiness only, and this is most akin to current public health strategies that focus on risks. The second program (called the naïve program) targets pleasure and riskiness randomly, and is most akin to a strategy where the policy maker has no prior knowledge about the behavioral effects of pleasure and riskiness perceptions. The third program (called the informed program) targets riskiness for smoking and for seatbelt nonuse and pleasure for drinking and for risky sex, and is most akin to a strategy where the policy maker is informed about the behavioral effects of pleasure and riskiness. The results of changing risk and pleasure perceptions as described in these three programs are shown in Table 5b; they provide us with an estimate of how behavior would change, on average, when all four behaviors are targeted. The informed program has the lowest odds ratio, followed by the naïve program, and then the current status quo riskiness-only program—suggesting that the informed program is the most effective in reducing unhealthy behavior. Formal tests of differences between the programs reveal that the informed program is statistically significantly better than both the riskiness-only program and the naïve program. A further test shows no statistical difference between the riskiness-only program and the naïve program. This highlights that current policy design is not performing better than chance in reducing unhealthy behavior.

## 4 Discussion

In this paper, we measured the respondents’ perceptions of the riskiness and the pleasure associated with four hypothetical unhealthy activities, and we examined how these perceptions are related to the probability that the respondents actually pursue very similar unhealthy behaviors in real life. Our findings add to the literature in different ways. We found that perceived pleasure predicted some behaviors but not others, and that perceived riskiness also predicted some behaviors but not others. This suggests that some behaviors are better explained by excessive pleasure seeking, while others are related more closely to insufficient risk avoidance. Furthermore, using a formal test of the difference in the effects of riskiness versus pleasure in predicting unhealthy behavior, we showed that the effect of pleasure was significantly greater than the effect of riskiness in predicting problem drinking and risky sex, but smoking showed the opposite pattern, and the effect of pleasure was indistinguishable from the effect of riskiness in predicting seat-belt nonuse. These results partly confirm prior findings that pleasure (or benefit) may be more important than riskiness (or cost) in determining unhealthy behavior, despite that the prior findings were from the U.S., Canada, and Australia while our study sample was from rural South Africa. Our results suggest that it is important not only to test for the significance of pleasure perceptions or riskiness perceptions in predicting behavior but also to test whether these perceptions have significantly different effects on behavior.

Our result that some behaviors are more related to pleasure seeking and others to risk-avoidance hints at the possibility that risky behaviors may need to be modeled using behavior-specific measures, such as the perceived pleasure and riskiness of each behavior. However, this does not imply that there is not a common risk attitude that drives risky behavior. In fact, our previous paper (Szrek et al., 2012) shows that a one-item risk taking propensity measure developed by Dohmen et al. (2011) predicts these unhealthy behaviors. In this current paper, we find that this one-item risk measure is no longer significant when we include behavior-specific pleasure and riskiness perceptions. This is similar to the finding by Maslowsky et al., (2011) that part of the effect of sensation seeking in determining unhealthy behaviors is mediated by risk-benefit perceptions. Overall, our findings in this paper and in previous work show that there are common elements across behaviors as well as behavior-specific elements to unhealthy risk taking.

### 4.1 Study limitations

We did not measure some other variables that may also drive behavior. In particular, the monetary costs of an unhealthy behavior were not assessed but could be even more important than riskiness and pleasure ratings in determining whether one pursues an unhealthy behavior or not. If monetary costs were more important, then increasing taxes to consumptive unhealthy behaviors (such as cigarettes or alcohol) may have greater policy impact (e.g., Grossman, 2004) than increasing riskiness perceptions, such as stiffer drunk driving laws (e.g., Freeman, 2007; Carpenter, 2004). Similarly, we use a single model to explain four different health behaviors that are clearly different from each other; in particular, smoking and drinking can be related to addiction, which would mean a different decision process. While the omission of the monetary cost and the use of a single framework are clear limitations of our study, we hope that future work does try to assess whether a simpler framework such as the parsimonious riskiness and pleasure ratings have very different findings than other more elaborate frameworks.

Our findings are also limited in that we studied one specific population. Our sample is that of individuals who originally went to get tested for HIV, so it may not be a representative sample even from the area we study. Concerns about selection bias are somewhat attenuated, however, by the fact that HIV tests are routinely done in South Africa for prenatal care and for various administrative reasons. Regardless, we urge researchers to examine the perceived pleasure and riskiness of these and other unhealthy behaviors in different populations.

Another limitation of our study is that the respondents in our sample may perceive riskiness associated with unprotected sex to be higher than the average population. This is because they received voluntary counseling and testing (VCT) for HIV two months prior to our study. Given that people in South Africa are already bombarded with information related to HIV, the additional information from VCT may not significantly alter respondent’s perceptions about the riskiness of sex without a condom. Some prior studies have also shown that high knowledge about HIV transmission risks does not necessarily translate to self-protection (Zetola et al. 2014). Nevertheless, this overall increase in perceived riskiness of unprotected sex may have contributed to us not being able to find an effect from riskiness. This also calls into question the effectiveness of any HIV prevention campaign that further focuses only on riskiness.

### 4.2 Policy implications

Our approach can also be useful in guiding the design of health promotion interventions. Because the effect of perceived riskiness and perceived pleasure in engaging in actual risky behavior differs by activity, interventions should also differ by activity. The current status quo focus on riskiness may no longer be the ideal strategy for public health interventions. Our results suggest that a strategy that utilizes information on the effectiveness of perceived pleasure and perceived riskiness, such as the “informed program” in Table 5b, is more effective than a public health intervention that focuses only on the risks of unhealthy behaviors. For instance, to decrease problem drinking, reducing perceived pleasure, say, by using disulfiram to induce unpleasant reactions when alcohol is ingested (Ntais et al., 2005) may work better than increasing perceived riskiness, for example by giving health warnings related to alcohol use (Babor et al., 2001). Also, increasing perceived riskiness of disease as a public health message may no longer be effective in increasing condom use, especially in a population already saturated with HIV-related messages (Green & Witte, 2006; Halperin, 2006). Efforts to increase the pleasure associated with sex with a condom may prove more effective. Partly for this reason, the topic of the Gates Foundation’s Grand Challenges Explorations Round 11 issued in March 2013 was to “Develop the Next Generation of Condom” with an aim to develop a condom that “enhance(s) pleasure” (Belluck, 2013).

In terms of reducing smoking or promoting seatbelt use, our results in Table 5a suggest that although higher riskiness perceptions do decrease pursuit of the unhealthy behaviors, increasing riskiness perception would not necessarily be more productive than decreasing pleasure perceptions. Public health messages have mostly focused on increasing riskiness perceptions, through, for example, presenting more gruesome pictures of cancer (Hammond et al. 2006), and by having school students try the Seatbelt Con-vincer to enable them to feel how risky it is to be without a seatbelt (Jessee, 1975; Robertson, 1978). Nevertheless, novel methods to decrease pleasure associated with smoking and seatbelt nonuse should also be developed and adopted. Restriction of nicotine in cigarettes is one such method, and an incessant beep that increases displeasure when a seatbelt is not worn is another.

### 4.3 Future research

We believe that the riskiness and pleasure framework offers a quick new tool to researchers and health promoters. This includes the application of riskiness and pleasure ratings in the economic evaluation of public health programs. As a simplified example, if the monetary cost to change the pleasure rating by one unit is the same as the monetary cost to change the riskiness rating by one unit, then a program that targets pleasure reduction will be more cost-effective in reducing problem drinking and risky sexual behavior than a program that tries to enhance riskiness perception. This is an over-simplified way of approaching an intervention design, and more thorough models and research must be performed. Nevertheless, our results point to the potential utility of the rapid assessment of riskiness and of pleasure as a preliminary study for public health program design and its evaluation. Once we understand how perceptions of riskiness and pleasure across populations and activities help to explain behavior, we will be prepared to develop more effective interventions.

Finally, although the bio-psycho-social downside risks associated with unhealthy behaviors are well established for smoking, heavy drinking, seatbelt nonuse, and risky sex without a condom, the pleasure mechanisms have only recently been revealed by neuroimaging. These neuroscience studies find that unhealthy activities trigger activation of the pleasure centers (including the nucleus accumbens) in the brain (e.g., Mitchell et al. 2012; David et al., 2005; Sabatinelli et al. 2007), with greater activation among people who pursue these activities in real life. Understanding why and the extent to which some people *enjoy* unhealthy activities should help in the design of ways to nudge people to healthier alternatives.

## Figures and Tables

**Table 1:** 

a: Means and correlations of pleasure ratings, riskiness ratings, and unhealthy behaviors.									
		Ratings (P = Pleasure; R = Riskiness)							
		Smoking		Drinking		No seatbelt		No condom	
	*Mean*	P	R	P	R	P	R	P	R
Ratings									
Smoking P	1.73								
Smoking R	6.19	−0.47**							
Drinking P	2.11	0.44**	−0.31**						
Drinking R	6.17	−0.22**	0.34**	−0.46**					
No seatbelt P	2.32	0.27**	−0.15**	0.33**	−0.15**				
No seatbelt R	6.07	−0.17**	0.31**	−0.19**	0.26**	−0.43**			
No condom P	1.74	0.46**	−0.23**	0.42**	−0.15**	0.27**	−0.18**		
No condom R	6.60	−0.21**	0.35**	−0.19**	0.31**	−0.10*	0.29**	−0.25**	–

Unhealthy behaviors									
Frequent smoking	0.11	0.09	−0.32**	0.12	−0.02	−0.09	−0.01	0.08	−0.04
Problem drinking	0.23	0.01	−0.12	0.30**	−0.13*	0.05	−0.02	0.16*	0.03
Seatbelt nonuse	0.24	−0.07	−0.04	0.08	−0.04	0.25**	−0.24**	0.12	−0.04
Not using condom	0.18	−0.03	−0.04	−0.02	−0.03	0.11	−0.04	0.09	0.05

b: Means and correlations between demographic variables and unhealthy behaviors					
		Unhealthy behaviors			
	*Mean*	Frequent smoking	Problem drinking	Seatbelt nonuse	Not using condom
Demographic variables					
Male gender	0.37	0.29**	0.28**	−0.03	−0.06
Age	31.87	0.08	−0.07	−0.17**	−0.02
Married or cohabiting	0.39	−0.00	−0.03	−0.04	−0.23**
Religion is very important	0.79	−0.04	−0.19**	−0.09	0.04
Numeracy (0 to 5)	2.21	0.21*	0.03	−0.01	0.11
Education		−0.29**	0.00	0.12	0.03
– less than primary	0.16				
– some primary	0.42				
– completed secondary	0.24				
– greater than secondary	0.18				
Income		−0.07	0.05	0.04	0.05
– R1000 or less per month	0.18				
– R1001 to R2000	0.21				
– R2001 to R4000	0.26				
– R4001 to R8000	0.18				
– R8001 or more	0.16				

Unhealthy behaviors					
Problem drinking	0.23	0.29**			
Seatbelt nonuse	0.24	−0.01	0.02		
Not using condom	0.18	−0.02	−0.04	0.07	–

c: Correlations between demographics and pleasure and riskiness ratings								
	Ratings (P = Pleasure; R = Riskiness)							
	Smoking		Drinking		No seatbelt		No condom	
Demographic variables	P	R	P	R	P	R	P	R
Male gender	0.08	−0.18**	0.14*	−0.07	−0.01	−0.11	0.16**	−0.07
Age	0.08	−0.03	−0.07	0.00	−0.03	0.04	0.00	−0.06
Married or cohabiting	0.10	0.05	0.01	−0.02	0.02	−0.04	0.05	−0.09
Religion is very important	−0.07	0.06	−0.19**	0.02	−0.10	0.03	−0.10	0.05
Numeracy (0 to 5)	−0.07	0.02	0.04	0.04	0.01	−0.03	−0.02	0.12*
Education	−0.16**	0.16**	−0.02	0.02	0.12*	−0.09	−0.08	0.21**
Income	−0.05	0.12*	−0.06	0.02	0.00	−0.02	−0.10	0.14**

d: Correlations of demographic variables						
Demographic variables	1	2	3	4	5	6
1. Male gender						
2. Age	−0.06					
3. Married or cohabiting	−0.08	0.31**				
4. Religion is very important	−0.08	0.15**	0.05			
5. Numeracy	0.15*	−0.18**	−0.20**	−0.07		
6. Education	0.02	−0.42**	−0.23**	−0.09	0.29**	
7. Income	0.12	−0.28**	0.01	−0.02	0.20**	0.36**

Note: Correlations between ratings are Spearman. Correlations between behaviors and ratings are rank biserial. N = 336.

** p<0.01;

* p<0.05.

Note: Correlations between behaviors and gender, marital status, and religiosity are Phi. Correlation between behaviors and age is point biserial. Correlations between behaviors and numeracy, education, and income are rank biserial.

** p<0.01;

* p<0.05.

Note: Correlations between gender, marital status, importance of religion, and ratings are rank biserial. Correlations between numeracy, education, income, and the ratings are Spearman.

** p<0.01;

* p<0.05.

Note: Correlations between gender, marital status, and religiosity are Phi. Correlations between numeracy, education, and income are Spearman. Correlations between age and gender, marital status, religiosity are point biserial. Correlations between gender, marital status, religiosity and numeracy, education, income are rank biserial.

** p<0.01;

* p<0.05.

**Table 2:** Mean ratings of pleasure (P) and riskiness (R).

Behavior in real life	N	Hypothetical activities	
	Smoking		
		P	R
Frequent smoker	38	2.03	5.37*
Not frequent smoker	298	1.69	6.30
	Binge drinking		
		P	R
Problem drinker	78	2.79**	5.95
Not problem drinker	258	1.90	6.24
	Not wearing seatbelt		
		P	R
Non-wearer of seatbelt	82	2.85**	5.54**
Wearer of seatbelt	254	2.15	6.24
	Not using condom		
		P	R
Condom non-user in risky sex	61	2.08*	6.70
Condom user or no risky sex	275	1.66	6.17

Note: The numbers in each cell represent the mean pleasure rating and riskiness rating in pursuing the activity, presented separately by self-reported unhealthy behavior. Significance of difference in ratings between those with vs. without self-reported unhealthy behavior are tested by interval regression of ratings on self-reported unhealthy behavior, while controlling for other covariates (gender, age, age squared, educational level, marital status, religiosity, income level, and numeracy).

** p<0.01;

* p<0.05.

**Table 3:** Odds ratios of pleasure and riskiness ratings in predicting real life unhealthy behavior.

	Unhealthy behavior pursued in real life			
	Frequent smoking	Problem drinking	Seatbelt nonuse	Not using condom
Pleasure rating	1.03 [0.70 to 1.53]	1.58** [1.21 to 2.08]	1.31* [1.02 to 1.68]	1.45** [1.10 to 1.90]
Riskiness rating	0.66* [0.48 to 0.91]	0.97 [0.75 to 1.26]	0.65** [0.50 to 0.84]	1.19 [0.84 to 1.70]

Note: Odds ratios [and 95% confidence intervals] were obtained from logistics regression. The dependent variable is the probability of whether the respondent pursues in real life a respective unhealthy behavior. The key explanatory variables are the standardized pleasure and riskiness ratings of a hypothetical unhealthy activity very similar to the real life unhealthy behavior. Other covariates include gender, age, age squared, educational level, marital status, religiosity, income level, and numeracy. See Appendix 2 for full regression models.

** p<0.01;

* p<0.05.

**Table 4:** Tests of differences of correlations of each behavior with pleasure (P) and riskiness (R).

Behavior in real life	N	t-statistic	Probability
Frequent smoking	349	−2.00	0.046
Problem drinking	348	2.47	0.014
Seatbelt nonuse	338	−0.69	0.490
Not using condom	350	2.24	0.026

Note: The table shows dependent correlations using the Steiger method. Sample includes all respondents with non-missing data on behavior, P, and R, despite missing values for other variables.

**Table 5:** 

a: Effectiveness of an intervention that decreases pleasure or increases riskiness by one unit			
Unhealthy behavior pursued in real life			
Frequent smoking	Problem drinking	Seatbelt nonuse	Not using condom
Each unhealthy behavior examined separately:			
Effectiveness of public health intervention that reduces P by one unit:			
0.97 [0.65 to 1.44]	0.63** [0.48 to 0.83]	0.76* [0.59 to 0.98]	0.69** [0.53 to 0.91]
Effectiveness of public health intervention that increases R by one unit:			
0.66* [0.48 to 0.91]	0.97 [0.75 to 1.26]	0.65** [0.50 to 0.84]	1.19 [0.84 to 1.70]
Whether difference between effectiveness of P vs. R interventions is significant or not:			
No	Yes*	No	Yes*

b. Effectiveness (odds ratios) of changing P or R for all four behaviors simultaneously		
	Odds ratios	Confidence interval
Riskiness-only program targeting R for all four unhealthy behaviors	0.50*	[0.25 to 0.97]
Naïve program targeting P and R randomly at 50% each	0.40**	[0.27 to 0.60]
Informed program targeting smoking R, drinking P, seatbelt R, and riskysex P	0.19**	[0.10 to 0.34]
Whether naïve program is more effective than riskiness-only program	No	
Whether informed program is more effective than riskiness-only program	Yes**	
Whether informed program is more effective than naïve program	Yes**	

Note: Odds ratios [and 95% confidence intervals] were obtained from logistics regression. The dependent variable is the probability of whether the respondent pursues in real life a respective unhealthy behavior. The key explanatory variables are the standardized pleasure and riskiness ratings of a hypothetical unhealthy activity very similar to the real life unhealthy behavior. Other covariates include gender, age, age squared, educational level, marital status, religiosity, income level, and numeracy.

** p<0.01;

* p<0.05.

Note: Odds ratios [and 95% confidence intervals] were obtained from post-estimation of the odds ratios in Table 5a.

** p<0.01;

* p<0.05.

## Footnotes

1 All survey measures are listed in full in Appendix 1. Here we summarize them.

2 Our results do not change if we consider instead how many drinks the respondent has on a single occasion (or how often he/she drinks) and similarly how often the respondent smokes.

3 We also asked questions related to risk taking propensity (Szrek et al., 2012), sensation seeking (Maslowsky et al., 2011), and time preference (Kirby & Marakovic, 1995), but because these measures are completely non-significant when perceived riskiness and perceived pleasure are used to model the unhealthy behaviors, we do not discuss them further in this paper. These measures are, however, listed in Appendix 1 for the interested reader. In Appendix 2, we also show regression results with these measures as additional control variables as well as a Wald test that compares the models with these measures to the models without them for each behavior.

4 See http://finzi.psych.upenn.edu/library/psych/html/r.test.html for how to implement this in R.

## Appendix 1: Key measures used in the study

### Self-reported actual risky behavior

#### Tobacco Use

*Tobacco use was defined as daily or almost daily use of any tobacco products, such as cigarettes, snuff, chewing tobacco, or cigars*.

TOB1 Do you currently use any tobacco products, such as cigarettes, snuff, chewing

tobacco, cigars, etc.?

1 = Yes
2 = No (Skip TOB2)

TOB2 In the past month, how often have you used tobacco products, such as cigarettes, snuff, chewing tobacco, cigars, etc.?

1 = Once or twice
2 = Weekly
3 = Almost daily
4 = Daily

#### Problem Drinking

*For problem drinking, we used the AUDIT-C (Bush et al., 1998), which consists of the first three questions from the Alcohol Use Disorder Identification Test questionnaire (Saunders et al., 1993). AUDIT-C measures consumption of alcohol (i.e., the frequency of drinking and the quantity consumed at a typical occasion) and the frequency of heavy episodic drinking. The third question from the original AUDIT questionnaire asks about frequency of having six or more drinks in one sitting (Saunders et al., 1993), which is equivalent to 60 grams of alcohol, based on 10 grams of alcohol per drink assumed in the AUDIT (Babor et al., 2001). Because one drink in South Africa on average contains 12 grams of alcohol (van Heerden and Parry 2001; Wolmarans et al. 1993), we modified this third question of AUDIT to five (instead of six) or more drinks in one sitting. Further, because later studies (e.g., Freeborn et al., 2000) suggest one fewer drink for women as a cutoff for sensible drinking, we modified this third question of AUDIT to four or more drinks in one sitting for women. The total AUDIT-C score is simply the sum of each of the AUDIT-C question’s scores. Men with a total AUDIT-C score equal to or greater than four and women with a total score equal to or greater than three (Bush et al., 1998; Gual et al., 2002) are then classified as problem drinkers*.

ALC1 How often did you have a drink containing alcohol in the past 12 months?

0 = Never (GO TO SX1)
1 = Once a month or less
2 = 2–4 times a month
3 = 2–3 times a week
4 = 4 or more times a week

ALC2 How many drinks containing alcohol do you have on a typical day when you are drinking?

0 = 1 or 2
1 = 3 or 4
2 = 5 or 6
3 = 7, 8, or 9
4 = 10 or more

ALC3 How often do you have (for men) five or more and (for women) four or more drinks in one sitting?

0 = Never
1 = Less than monthly
2 = Monthly
3 = Weekly
4 = Daily or almost daily

#### Seatbelt Non-use

*Seatbelt use was assessed by asking the respondent if a seat belt was used the last time the respondent sat in the front seat of a car*.

SB1 Did you wear a seatbelt the last time you sat in the front seat of a car?

1 = Yes
2 = No

#### Risky Sex Without a Condom

*A respondent was classified as someone who had risky sex if (i) the respondent was not married/cohabitating and did not use a condom during the most recent sexual intercourse or (ii) the respondent was married/cohabiting, but had more than one partner in the last 12 months and did not use a condom during last sex*.

SX1 How many sexual partners did you have during the past 12 months?

(NUMBER)

SX2 Did you use a condom the last time you had sex?

1 = Yes
2 = No
3 = Virgin (never had sex)

### Perceived riskiness and perceived pleasure

#### Perceived riskiness

“With 1 representing not at all risky and 7 being extremely risky, how risky do you think it is to: *Drink heavily on weekends*.”

#### Perceived pleasure

“With 1 representing no pleasure at all and 7 representing extreme pleasure, how much pleasure do you derive from: *Drinking heavily on weekends*.”

*The italics was replaced by the other health risk behaviors*.

*Health risk behaviors: smoke half a pack of cigarettes a day, sit in the front seat of a car without a seatbelt, and have sex with a new partner without a condom*.

### One-item risk attitude measure

Are you generally a person who is fully prepared to take risks or do you generally always try to avoid taking risks? Using any number between 1 to 7, with 1 indicating that you generally “do everything possible to avoid taking risks” and 7 indicating that you are generally “fully prepared to take risks”, please tell me which number represents you best.

*The original 0 to 10 scale from Dohmen et al. (2011) was modified to a 1 to 7 scale to make it consistent with the other questions in the questionnaire*.

### Sensation seeking

*Steinberg et al. (2008) six-item Sensation Seeking Scale*.

All responses 1=Agree or 2=Disagree.

1. I like to have new and exciting experiences and sensations even if they might be a little scary to me.
2. I like to do certain things just for the thrill of it.
3. I sometimes like to do things that are a little frightening.
4. I will try anything once.
5. I sometimes do “crazy” things just for fun.
6. I tend to enjoy “wild” uninhibited parties.

### Time-Preference

*We asked 10 time-preference trade-off questions modeled after Kirby and Marakovic (1995). We changed the time periods to take advantage of the fact that respondents were returning to the clinics in 2 months time. (This made the questions more believable and real.)*

1. Would you prefer to receive R45 today or to receive R50 in 2 months from today?
  1 = R45 today
  2 = R50 in 2 months
2. R40 today or R50 in 2 months?
  1 = R40 today
  2 = R50 in 2 months
3. R5 today or R50 in 2 months?
  1 = R5 today
  2 = R50 in 2 months
4. R20 today or R50 in 2 months?
  1 = R20 today
  2 = R50 in 2 months
5. R10 today or R50 in 2 months?
  1 = R10 today
  2 = R50 in 2 months
6. R30 today or R50 in 2 months?
  1 = R30 today
  2 = R50 in 2 months
7. R15 today or R50 in 2 months?
  1 = R15 today
  2 = R50 in 2 months
8. R35 today or R50 in 2 months?
  1 = R35 today
  2 = R50 in 2 months
9. R1 today or R50 in 2 months?
  1 = R1 today
  2 = R50 in 2 months
10. R25 today or R50 in 2 months?
  1 = R25 today
  2 = R50 in 2 months

### Demographics

Gender (filled in by enumerator)

0=Female
1=Male

HQ1 What is your age?

(AGE IN YEARS)

HQ2 What is your marital status?

1 = Single
2 = Living together, but not married
3 = Married or Customary marriage
4 = Widowed
5 = Separated or Divorced

HQ3 What level of education did you complete?

1 = None
2 = Some primary
3 = Standard 5/ Grade 7 (End Primary)
4 = Some secondary
5 = Standard 10/ Grade 12 (End Secondary)
6 = Some Tertiary (Std 10 & some tertiary)
7 = Tertiary (Std 10 & Degree or Diploma)

DU2 How important is religion to you in your life?

1 = Not important at all
2 = Slightly important
3 = Somewhat important
4 = Important
5 = Very important
6 = No Religion or Not Applicable

W6 What is your average monthly household income before tax (including all salaries, piece jobs, and grants)?

1 = No income
2 = Less than R500 per month
3 = R501 to R1 000 per month
4 = R1 001 to R2 000 per month
5 = R2 001 to R4 000 per month
6 = R4 001 to R8 000 per month
7 = R8 001 to R16 000 per month
8 = R16 000 to R32 000 per month
9 = More than R32 000 per month

#### Numeracy questions

*Note: CQ1 was asked to relax the respondent and was not incorporated in the numeracy score. Scale ranged from 0 to 5, as the number of correct answers on CQ2 to CQ6*.

CQ1 Which is heavier: 100 kg of feathers or 100 kg of steel?

1 = 100kg Feathers
2 = 100kg Steel
3 = Same

CQ2 Tomatoes at Checkers sell for 21 cents per kg. What will 4 kg of tomatoes cost?

CQ3 A boy is 6 years old and his sister is twice as old. When the boy is 10 years old, what will be the age of his sister?

CQ4 A patch of weed in a garden grows and doubles in size every day. If it takes the weed 48 days to cover the whole garden, how many days does it take the weed to cover half the garden?

CQ5 True or False: Two chickens and two dogs have a total of 14 legs.

1 = True
2 = False

CQ6 Thandiwe needs 13 bottles of water from the store. She can only carry 3 at a time. What is the minimum number of trips she needs to make to the store?

## Appendix 2: Odds ratios of pleasure and riskiness ratings and other covariates in predicting real life unhealthy behavior

**Table:**

	Frequent smoking	Problem drinking	Seatbelt nonuse	Not using condom	Frequent smoking	Problem drinking	Seatbelt nonuse	Not using condom
Smoking P	1.031 [0.696,1.527]				0.997 [0.683,1.457]			
Smoking R	0.660* [0.478,0.911]				0.676* [0.482,0.950]			
Drinking P		1.583*** [1.206,2.078]				1.569** [1.188,2.072]		
Drinking R		0.972 [0.750,1.260]				1.02 [0.779,1.335]		
No seatbelt P			1.310* [1.019,1.684]				1.296* [1.001,1.679]	
No seatbelt R			0.649** [0.501,0.840]				0.673** [0.520,0.870]	
No condom P				1.447** [1.104,1.895]				1.449** [1.105,1.900]
No condom R				1.193 [0.836,1.702]				1.21 [0.849,1.725]
Male gender	7.455*** [3.362,16.53]	3.649*** [2.032,6.552]	0.777 [0.443,1.361]	0.490* [0.249,0.964]	7.996*** [3.426,18.66]	3.618*** [2.001,6.543]	0.741 [0.422,1.301]	0.509 [0.257,1.006]
Age	1.04 [0.858,1.261]	0.961 [0.829,1.115]	0.936 [0.768,1.140]	1.071 [0.915,1.253]	1.04 [0.864,1.253]	0.954 [0.826,1.103]	0.929 [0.769,1.122]	1.077 [0.920,1.262]
Age squared	1 [0.997,1.002]	1 [0.999,1.002]	1 [0.997,1.003]	0.999 [0.997,1.001]	1 [0.997,1.002]	1.001 [0.999,1.002]	1 [0.998,1.003]	0.999 [0.997,1.001]
Education: some primary	0.172** [0.0546,0.541]	0.754 [0.313,1.819]	0.296* [0.104,0.841]	0.295* [0.0978,0.888]	0.153** [0.0444,0.527]	0.809 [0.312,2.094]	0.295* [0.104,0.835]	0.316* [0.102,0.980]
Education: completed secondary	0.144** [0.0371,0.558]	0.902 [0.332,2.453]	0.498 [0.167,1.486]	0.235* [0.0687,0.804]	0.124** [0.0299,0.517]	0.951 [0.329,2.748]	0.492 [0.164,1.477]	0.243* [0.0687,0.860]
Education: greater than secondary	0.0821** [0.0173,0.388]	0.551 [0.195,1.560]	0.554 [0.168,1.827]	0.376 [0.0983,1.435]	0.0670** [0.0127,0.353]	0.537 [0.182,1.587]	0.518 [0.156,1.724]	0.368 [0.0939,1.444]
Married or cohabiting	0.864 [0.354,2.107]	0.974 [0.523,1.816]	0.906 [0.499,1.648]	0.162*** [0.0727,0.362]	0.857 [0.326,2.256]	1.018 [0.537,1.929]	0.976 [0.530,1.800]	0.169*** [0.0760,0.375]
Numeracy	1.724* [1.052,2.823]	0.915 [0.676,1.238]	0.889 [0.673,1.174]	1.155 [0.814,1.638]	1.764* [1.056,2.948]	0.905 [0.667,1.227]	0.879 [0.662,1.166]	1.155 [0.810,1.648]
Religion is very important	0.734 [0.290,1.857]	0.419** [0.221,0.795]	0.712 [0.380,1.336]	1.452 [0.662,3.188]	0.78 [0.300,2.029]	0.439* [0.224,0.860]	0.755 [0.401,1.422]	1.437 [0.665,3.104]
Income: R1001 to R2000	1.146 [0.313,4.199]	1.63 [0.626,4.248]	1.782 [0.699,4.546]	4.169* [1.245,13.97]	1.077 [0.263,4.400]	1.545 [0.570,4.190]	1.635 [0.643,4.157]	4.187* [1.268,13.82]
Income: R2001 to R4000	0.92 [0.273,3.103]	1.174 [0.461,2.986]	1.814 [0.743,4.431]	4.769* [1.452,15.67]	0.86 [0.238,3.108]	1.067 [0.407,2.799]	1.577 [0.649,3.833]	4.594* [1.421,14.85]
Income: R4001 to R8000	0.68 [0.158,2.921]	1.428 [0.508,4.009]	1.089 [0.378,3.132]	4.289* [1.143,16.09]	0.682 [0.147,3.168]	1.336 [0.463,3.856]	0.95 [0.328,2.748]	4.108* [1.103,15.30]
Income: R8001 or more	1.386 [0.346,5.559]	1.244 [0.426,3.636]	1.093 [0.371,3.218]	3.514 [0.822,15.02]	1.369 [0.336,5.580]	1.198 [0.417,3.441]	1.02 [0.341,3.048]	3.355 [0.807,13.95]
Risk trait					0.986 [0.796,1.221]	1.096 [0.944,1.273]	1.085 [0.957,1.229]	1.052 [0.917,1.206]
Sensation seeking					1.151 [0.888,1.492]	1.109 [0.904,1.360]	1.153 [0.960,1.383]	1.001 [0.812,1.234]
Time preference					0.547 [0.122,2.449]	0.963 [0.311,2.986]	1.067 [0.402,2.830]	0.461 [0.136,1.561]

N	336	336	336	336	336	336	336	336
Pseudo R2	0.249	0.141	0.121	0.139	0.257	0.151	0.135	0.146
Wald test Chi-square					2.1	4.35	5.38	1.95
p-value					0.5527	0.2257	0.146	0.5839

Note: 95% confidence intervals for the odds ratios in brackets;

* p<0.05,

** p<0.01,

*** p<0.001.

The Wald test compares the regressions 5 through 8, that include the risk trait, sensation seeking and time preference, with the corresponding regressions in columns 1 through 4.

## References

[R1] Ajzen I (1991). The theory of planned behavior. Organizational Behavior and Human Decision Processes.

[R2] Babor TF, Higgins-Biddle JC, Saunders JB, Monteiro MG (2001). AUDIT: The Alcohol Use Disorders Identification Test, Guidelines for use in primary care.

[R3] Bauman KE (1980). Predicting adolescent drug use: Utility structure and marijuana.

[R4] Bauman KE, Fisher LA, Bryan ES, Chenoweth RL (1984). Antecedents, subjective expected utility, and behavior: A panel study of adolescent cigarette smoking. Addictive Behaviors.

[R5] Bauman KE, Fisher LA, Bryan ES, Chenoweth RL (1985). Relationship between subjective expected utility and behavior: A longitudinal study of adolescent drinking behavior. Journal of Studies on Alcohol and Drugs.

[R6] Bauman KE, Udry JR (1981). Subjective expected utility and adolescent sexual behavior. Adolescence.

[R7] Belluck P (2013). Getting men to want to use condoms.

[R8] Benthin A, Slovic P, Severson H (1993). A psychometric study of adolescent risk perception. Journal of Adolescence.

[R9] Bush K, Kivlahan DR, McDonell MB, Fihn SD, Bradley KA (1998). The audit alcohol consumption questions (AUDIT-C), an effective brief screening test for problem drinking. Archives of Internal Medicine.

[R10] Carpenter C (2004). How do zero tolerance drunk driving laws work?. Journal of Health Economics.

[R11] David SP, Munaf`o MR, Johansen-Berg H, Smith SM, Rogers RD, Matthews PM, Walton RT (2005). Ventral striatum/nucleus accumbens activation to smoking-related pictorial cues in smokers and nonsmokers: a functional magnetic resonance imaging study. Biological Psychiatry.

[R12] Dhami MK, Garcia-Retamero R (2012). Spanish young adults’ perceptions of the costs and benefits of risky driving behaviors. The Spanish Journal of Psychology.

[R13] Dhami MK, Mandel DR (2012). Forecasted risk taking in youth: evidence for a bounded-rationality perspective. Synthese.

[R14] Dhami MK, Mandel DR, Garcia-Retamero R (2011). Canadian and Spanish youths’ risk perceptions of drinking and driving, and riding with a drunk driver. International Journal of Psychology.

[R15] Dohmen T, Falk A, Huffman D, Sunde U, Schupp J, Wagner GG (2011). Individual risk attitudes: Measurement, determinants, and behavioral consequences. Journal of the European Economic Association.

[R16] Edwards W (1954). The theory of decision making. Psychological Bulletin.

[R17] Edwards W (1961). Behavioral decision theory. Annual Review of Psychology.

[R18] Freeborn DK, Polen MR, Hollis JF, Senft RA (2000). Screening and brief intervention for hazardous drinking in an HMO: effects on medical care utilization. The Journal of Behavioral Health Services and Research.

[R19] Freeman DG (2007). Drunk driving legislation and traffic fatalities: new evidence on bac 08 laws. Contemporary Economic Policy.

[R20] Gelman A, Stern H (2006). The difference between “significant” and “not significant” is not itself statistically significant. American Statistician.

[R21] Green EC, Witte K (2006). Can fear arousal in public health campaigns contribute to the decline of hiv prevalence?. Journal of Health Communication.

[R22] Grossman M (2004). Individual behaviors and substance use: the role of price. Technical report 10948, National Bureau of Economic Research.

[R23] Gruber J (2001). Risky behavior among youths: An economic analysis.

[R24] Gual A, Segura L, Contel M, Heather N, Colom J (2002). Audit-3 and audit-4: effectiveness of two short forms of the alcohol use disorders identification test. Alcohol and Alcoholism.

[R25] Halperin DT (2006). The controversy over fear arousal in AIDS prevention and lessons from Uganda. Journal of Health Communication.

[R26] Hammond D, Fong G, McNeill A, Borland R, Cummings KM (2006). Effectiveness of cigarette warning labels in informing smokers about the risks of smoking: Findings from the international tobacco control (ITC) four country survey. Tobacco control.

[R27] Janis IL, Mann L (1977). Decision making: A psychological analysis of conflict, choice, and commitment.

[R28] Jessee WK (1975). A survey to determine the influence of the seatbelt convincer on seatbelt usage by the general public. Ed.D. Dissertation.

[R29] Kirby KN, Maraković NN (1995). Modeling myopic decisions: Evidence for hyperbolic delay-discounting within subjects and amounts. Organizational Behavior and Human Decision Processes.

[R30] Maslowsky J, Buvinger E, Keating DP, Steinberg L, Cauffman E (2011). Cost-benefit analysis mediation of the relationship between sensation seeking and risk behavior among adolescents. Personality and Individual Differences.

[R31] Mitchell JM, O’Neil JP, Janabi M, Marks SM, Jagust WJ, Fields HL (2012). Alcohol consumption induces endogenous opioid release in the human orbitofrontal cortex and nucleus accumbens. Science Translational Medicine.

[R32] Moore S, Gullone E (1996). Predicting adolescent risk behavior using a personalized cost-benefit analysis. Journal of Youth and Adolescence.

[R33] Ntais C, Pakos E, Kyzas P, Ioannidis JPA (2005). Benzodiazepines for alcohol withdrawal. Cochrane Database Syst Rev.

[R34] Parsons JT, Siegel AW, Cousins JH (1997). Late adolescent risk-taking: Effects of perceived benefits and perceived risks on behavioral intentions and behavioral change. Journal of Adolescence.

[R35] Prochaska JO, Velicer WF, Rossi JS, Goldstein MG, Marcus BH, Rakowski W, Fiore C, Harlow LL, Redding CA, Rosenbloom D (1994). Stages of change and decisional balance for 12 problem behaviors. Health Psychology.

[R36] Robertson LS (1978). Automobile seat belt use in selected countries, states and provinces with and without laws requiring belt use. Accident Analysis & Prevention.

[R37] Rosenstock IM, Strecher VJ, Becker MH (1988). Social learning theory and the health belief model. Health Education & Behavior.

[R38] Sabatinelli D, Bradley MM, Lang PJ, Costa VD, Versace F (2007). Pleasure rather than salience activates human nucleus accumbens and medial prefrontal cortex. Journal of Neurophysiology.

[R39] Saunders JB, Aasland OG, Babor TF, de la Fuente JR, Grant M (1993). Development of the alcohol use disorders identification test (AUDIT): Who collaborative project on early detection of persons with harmful alcohol consumption-II. Addiction.

[R40] Siegel AW, Cousins JH, Rubovits DS, Parsons JT, Lavery B, Crowley CL (1994). Adolescents’ perceptions of the benefits and risks of their own risk taking. Journal of Emotional and Behavioral Disorders.

[R41] Steiger JH (1980). Tests for comparing elements of a correlation matrix. Psychological Bulletin.

[R42] Steinberg L, Albert D, Cauffman E, Banich M, Graham S, Woolard J (2008). Age differences in sensation seeking and impulsivity as indexed by behavior and self-report: evidence for a dual systems model. Developmental Psychology.

[R43] Szrek H, Chao L-W, Ramlagan S, Peltzer K (2012). Predicting (un) healthy behavior: A comparison of risk-taking propensity measures. Judgment and Decision Making.

[R44] Velicer WF, DiClemente CC, Prochaska JO, Brandenburg N (1985). Decisional balance measure for assessing and predicting smoking status. Journal of Personality and Social Psychology.

[R45] van Heerden I, Parry C (2001). If you drink alcohol, drink sensibly. South African Journal of Clinical Nutrition.

[R46] Weber EU, Blais A-R, Betz NE (2002). A domain-specific risk-attitude scale: Measuring risk perceptions and risk behaviors. Journal of Behavioral Decision Making.

[R47] Wolmarans P, Langenhoven M, Faber M (1993). Food facts and figures.

[R48] Zetola NM, Modongo C, Olabiyi B, Ramogola-Masire D, Collman RG, Chao L-W (2014). Examining the relationship between alcohol use and high-risk sex practices in a population of women with high hiv incidence despite high levels of HIV-related knowledge. Sexually Transmitted Infections.

